# Supplementary material for: Health conditions of potential risk for severe Covid-19 in institutionalized elderly people
Source: PLoS One. 2021 Jan 14;16(1):e0245432. doi: 10.1371/journal.pone.0245432 (PMC7808625; doi:10.1371/journal.pone.0245432)
Supplement: Attachment — Submitted filename: Response to Reviewers.docx [file pone.0245432.s003.docx]

Mayara Priscilla Dantas Araújo

Federal University of Rio Grande do Norte

Natal, Brazil

mayaraaraujonutri@gmail.com

December 31st, 2020

Dear PLoS ONE,

We would like to express our sincere gratitude to the editor and the reviewer for their generous comments on our manuscript titled “Association between nutritional status and risk factors for COVID-19 in institutionalized elderly people”.

We have revised the manuscript to address all the concerns raised by the editor and the reviewer. And we believe that the manuscript is now suitable for publication in PLOS One.

Please find below our responses to the issues raised.

Thank you,

Mayara Priscilla Dantas Araújo

Corresponding Author

On behalf of all authors.

- Please provide further details on sample size and power calculations.

*Response:* In this study, it was used the population of elderly living in the one of the eight Long-Term Care Facilities for the Elderly (LTCF) in the metropolitan region of Natal, Rio Grande do Norte, Brazil. For this reason, in this study was not necessery to performe a sample calculation.

- Please clarify the sources of funding (financial or material support) for your study. List the grants or organizations that supported your study, including funding received from your institution.

*Response:* The project was funded by the International Network for Research on Vulnerability, Health, Safety and Quality of Life of the Elderly: Brazil, Portugal and Spain (Edital 01/2020/UFRN), GVT is a productivity fellow by the National Council for Scientific and Technological Development (CNPq) [process 309213/2017-7] and MPDA is a fellow by the Coordenação de Aperfeiçoamento de Pessoal de Nível Superior - Brazil (CAPES) - Finance Code 001.

- Is the manuscript technically sound, and do the data support the conclusions?

The manuscript must describe a technically sound piece of scientific research with data that supports the conclusions. Experiments must have been conducted rigorously, with appropriate controls, replication, and sample sizes. The conclusions must be drawn appropriately based on the data presented.

Reviewer #1: No

*Response:* We identified errors that compromised the understanding of our study. We made the changes in the manuscript in order to improve its understanding. With this, we realized the need to rewrite the objective and the conclusion in order to ensure that our study meets the comments of the reviewer and the journal.

5. Review Comments to the Author

Reviewer #1: This manuscript included the epidemiological data of a specific population in the capital of a Brazilian state. For the analysis, individuals were selected from a period when the COVID-19 pandemic did not exist. Individual characteristics may have changed over the period between data collection and the outbreak of the epidemic. There is a gap of approximately one year between data collection and the start of the pandemic. In addition, it was not clear why the data found were considered risk factors for severe forms of COVD-19 infection, since there are no reports that the study subjects developed the disease. I do not consider the data in the present study to be relevant, since a direct relationship between risk factors and the development of the disease in its various forms has not been demonstrated.

*Response:* Although the Covid-19 pandemic did not exist when the study data were collected, we observed, based on the literature, that the epidemiological profile of the elderly analyzed resembles that of the elderly domiciled and that of the world population. Moreover, the nutritional profile of the elderly is also maintained, with weight reduction as age progresses. Thus, we understand that the analyzed data can provide support to the planning of protection actions for this public because their health conditions can contribute to the worsening of Covid-19 in case of infection and the deleterious effects of this disease in these institutions already reported worldwide.

We observed that in some parts we did not make ourselves understood correctly. The writing made the reader understand that the presence of risk factors would lead to the development of the disease, instead of these risk factors can lead to the development of the severe form of Covid-19 in case of infection, as established in the literature. In this way, we make the changes throughout the text for a complete understanding of our results and conslusion.
